# HSV-2 Increases TLR4-Dependent Phosphorylated IRFs and IFN-β Induction in Cervical Epithelial Cells

**DOI:** 10.1371/journal.pone.0094806

**Published:** 2014-04-10

**Authors:** Hongya Liu, Kai Chen, Wenqiang Feng, Juanjuan Guo, Hui Li

**Affiliations:** 1 State Key Laboratory of Virology, Institute of Medical Virology, Wuhan University School of Medicine, Wuhan, Hubei, China; 2 Shengzhen R&D Center of State Key Laboratory of Virology, Wuhan University Shenzhen Institute, Shenzhen, Guangdong, China; 3 Central Blood Station of Rizhao, Rizhao, Shandong, China; Georgetown University, United States of America

## Abstract

Our previous studies demonstrated that HSV-2 infection up-regulates TLR4 expression and induces NF-kB activity, thereby facilitating innate immune response in human cervical epithelial cells. This process requires involvement of TLR4 adaptors, Mal and MyD88. In the current study, we found that HSV-2 infection increases levels of phosphoryalted IRF3 and IRF7, then regulating expression of type I IFN. As expected, these changes induced by HSV-2 infection depended upon TLR4. Knockdown of TRIF and/or TRAM by siRNAs indicated that TRIF/TRAM might be involved in expression of IFN-β. Our results demonstrate for the first time that IRF3 and IRF7 are both involved in inducing TLR4-dependent IFN-β expression in response to HSV-2 in its primary infected genital epithelial cells. Thus, TLR4-Mal/MyD88 and TLR4-TRIF/TRAM signaling may synergize and/or cooperate in innate immune response of cervical epithelial cells to HSV-2 infection.

## Introduction

Herpes simplex virus type 2 (HSV-2) infection causes genital ulcer disease and is now considered a major risk factor for the acquisition, transmission and progression of human immunodeficiency virus type 1 (HIV-1) [1]. Recently HSV-2 has been recognized as a potentially important factor in the pathogenesis of Kaposi's sarcoma (KS) [2]. HSV-2 infects the genital epithelium and can be transmitted to the central nervous system to establish life-long latent infection. Current treatments with anti-viral therapy are commonly used to control re-activation of HSV-2. However, these medications do not eliminate latent virus [3]. The increasing incidence and prevalence of HSV-2 and its association with significant morbidity and mortality has urged us to uncover its fundamental mechanisms.

The genital mucosa is the first line of defense against sexually transmitted pathogens and plays a crucial role in innate immunity and adaptive immunity as well[4]. HSV-2 primarily infects genital epithelium and replicates within the vaginal keratinocytes [5]. Recently, a Canadian group investigated the susceptibility of primary human female genital epithelial cells to HSV-2 using an ex vivo culture model [6]. By using TLR ligands, they assessed the anti-viral activity of human female genital epithelium in response to HSV-2[7] and the role of HSV-2 virion host shutoff protein on innate dsRNA antiviral pathways in human vaginal epithelial cells [8]. But to date, little is known about the innate immune pathways of human genital epithelial cells in response to HSV-2 infection.

Although it has been shown that multiple Toll-like receptors (TLRs) are involved in recognition of different HSV strains and contribute to the immune response to HSV infection[7], [9], [10], [11], [12], [13], [14], [15]. Most of these studies have used immuno-competent cells or mice model. There appears discrepancy while assessing the role of NK cells, conventional dendritic cells (cDCs) and plasmacytoid DCs (pDCs) in HSV-2 infection [4]. One of the assignable facts is that HSV-2 does not directly infect DCs [16]. IFN-β production or IFN-sensing pathways have been shown pronounced differences between human DCs and genital epithelial cells [17]. It has been realized that the experimental immunology studies should link directly to the diseases caused by HSV in humans [18]. Therefore, our recent study has focused on the human primary target cells of HSV-2 and established an *in vitro* HSV-2 acute infection model with Human Cervical Epithelial (HCE) cells to investigate the role of TLRs-mediated innate immune response to HSV-2 [19].

IFN-beta plays a critical role in antiviral activity during the initial HSV-2 infection of genital epithelium [17]. Different cell types are likely to have their own preferred pathways to induce type I IFN in response to different viruses. The interferon-regulatory factor (IRF) family of transcription factors are differentially activated and function as important mediators of IFNs, for example, IRF8 and IRF3 cooperatively regulate IFN-β induction in human monocytes to respond Sendai Virus [8], [20], [21], [22], [23]. But there is discrepancy if IRF7 is required for the induction of IFN-β upon virus infection. It is worthwhile to investigate the key IRF family members in regulating IFN-β expression downstream of TLRs in response to HSV-2.

We have shown that HSV-2 infection up-regulates TLR4 expression and activates NF-kB, and over-expression of TLR4/MD2 augments viral-induced NF-kB activation. In the current study, we found that HSV-2 infection activates TLR4-dependent IRF3 and IRF7 which are key players in regulating TLR-mediated type I IFN expression. The two adaptor molecules TRIF and TRAM of TLRs signaling pathways might also be involved in this TLR4-mediated innate immune signaling. Our results demonstrate for the first time that IRF3 and IRF7 are both involved in inducing TLR4-dependent IFN-β expression in response to HSV-2 in its primary infected genital epithelial cells.

## Results

### HSV-2 infection induced production of IFN-β in HCE cells is TLR4-dependent

**Figure 1:**
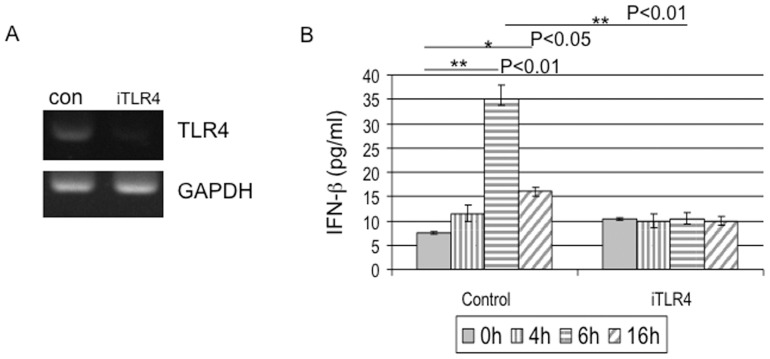
HSV-2 induced production of IFN-β in HCE cells is TLR4-dependent. (A) Knockdown of TLR4 expression. The HCE cells were transfected with shRNA-TLR4 plasmid (refer to iTLR4) or scrambled shRNA control. Forty-eight hours after transfection, the total RNAs were collected and the expression of TLR4 was analyzed by RT-PCR. The expression of GAPDH was used as the internal control. The experiment was repeated three times, the representative images from one experiment are shown. (B) TLR4-dependent IFN-β expression by HSV-2 infection. HCE cells were transfected with shRNA-TLR4 or vector control. At 48 h after transfection, HCE cells were infected with HSV-2 at 3 MOI or mock-infected. Cell-free supernatants were collected at 0 h, 4 h, 6 h, 16 h post-infection for measurement of IFN-β secretion by ELISA analysis. Each assay was performed in triplicate. Results are reported as means ± SD. ** indicates p<0.01 and * indicates p<0.05.

In previous study we established an *in vitro* acute HSV-2 infection model with human cervical epithelial (HCE) cells for the study of innate immune response [19]. We found that the activation of NF-kB is required for the production of IFN-β induced by HSV-2 in HCE cells. More importantly, TLR4 potentially contributes to the innate immune response to HSV-2 infection in its natural host cells [19]. Type I IFNs are the major factors in host mucosal defense to HSV-2 infection [24]. To further explore whether TLR4 plays a role in the production of IFN-β induced by HSV-2, we knock-down TLR4 expression in HCE cells by shRNA construct specific target for TLR4 (refer to iTLR4). We confirmed the knock-down efficiency of iTLR4 by RT-PCR (Figure 1A). HCE cells were transfected with vector or iTLR4 for 48 h and then infected with HSV-2 or mock-infected. At the indicated time points post-infection (p.i.), we collected the supernatants from cell cultures and measured the secretion of IFN-β (Figure 1B) by ELISA assay. The basal IFN-β was 7.50±0.25 pg/ml from mock infected HCE cells. HSV-2 infection induced the expression of IFN-β and the secretion value was increased to 35.00±3.00 pg/ml at 6 h p.i.(p<0.01) and 16.00±1.00 pg/ml at 16 h p.i. (p<0.05) (Figure 1B). The production and secretion of INF-β shows an accumulation peak value at 6 h p.i. and declines at 16 p.i.. This indicates the time-dependent immune responses of HCE cells to viral infection. While the basal level of IFN-β was elevated to 10.50±0.25 pg/ml from mock infected and iTLR4 transfected cells, knock-down TLR4 decreased HSV-2 induced IFN-β to the basal level (10.50±1.25 pg/ml) at 6 h p.i. (p<0.01) (Figure 1B). These data suggested that TLR4 is critical for the expression and secretion of IFN-β induced by HSV-2 infection in HCE cells.

### TLR4 mediates the phosphorylation of IRF3 in response to HSV-2

**Figure 2:**
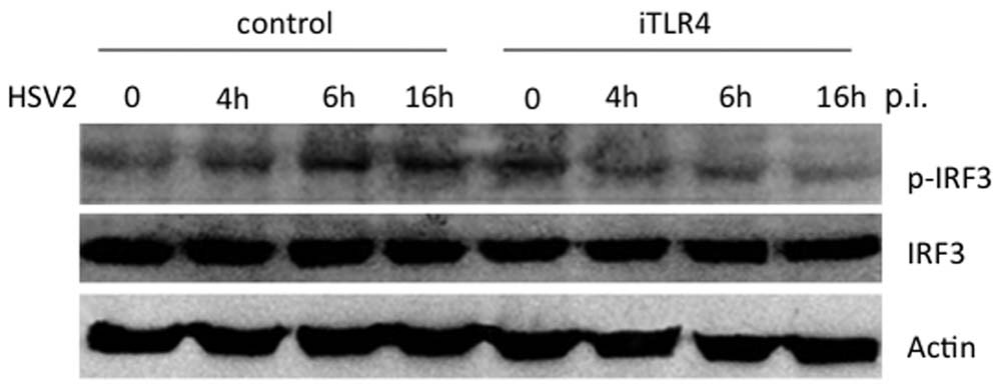
TLR4 mediates the phosphorylation of IRF3 in response to HSV-2. HCE cells were transfected with scrambled shRNA control or iTLR4. Forty-eight hours after transfection, cells were infected with HSV-2 at 3 MOI or mock-infected. Cell lysates were collected at 0 h, 4 h, 6 h, 16 h post-infection and analyzed by Western blotting with antibodies against p-IRF3, total IRF3 and Actin. Knockdown of TLR4 decreased the phosphorylation of IRF3 by HSV2 infection. The experiment was repeated three times, the representative images from one experiment are shown.

The interferon-regulatory factor (IRF) family of transcription factors has been shown their crucial roles in immuno-regulation by TLRs [25]. Depending on the nature of the pathogen and cell type, IRFs are differentially activated and function as important mediators of the innate immune response [25]. IRF3 is the key regulator of type I IFN gene expression elicited by viral infection [22]. We have shown that HSV-2 infection activates TLR4-dependent production of IFN-β in HCE cells. We wanted to assess if TLR4 mediates the activation of IRF3 to regulate expression of IFN-β in response to HSV-2 infection. We transfected HCE cells with scrambled shRNA control or iTLR4 and then infected cells with HSV-2 at 3 MOI or mock-infected. We harvested cell lysates from above cells at the indicated time points post-infection (p.i.) for Western blot analysis. As shown in Figure 2, IRF3 was phosphorylated at 4 h after HSV-2 infection. The level of phosphorylated IRF3 increased at 6 h p.i. and remained the elevated level at 16 h p.i.. While in the HCE cells where TLR4 was knockdown with iTLR4, the basal level of phosphorylated IRF3 increased compared to the normal uninfected HCE cells. However, HSV-2 induced phosphorylation of IRF3 was abolished by knockdown of TLR4 at 4 h, 6 h and 16 h p.i. (Figure 2). During the time-course of viral infection, the total IRF3 remained unchanged (Figure 2) and actin was used as the internal loading control. These data suggested that TLR4 indeed mediates the phosphorylation and activation of IRF3 which thereafter transcribes the gene expression of IFN-β upon HSV-2 stimulation in HCE cells.

### TLR4 mediates the phosphorylation of IRF7 in response to HSV-2

**Figure 3:**
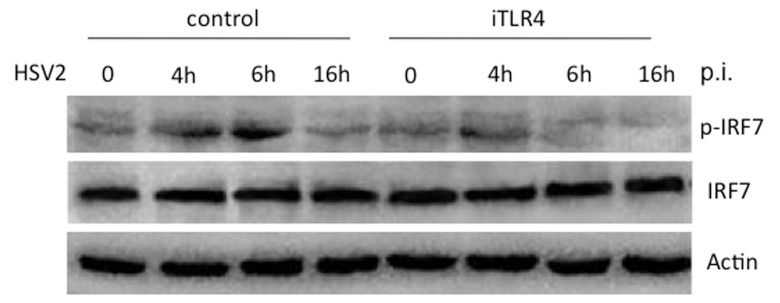
TLR4 mediates the phosphorylation of IRF7 in response to HSV-2. HCE cells were transfected with scrambled shRNA control or iTLR4. Forty-eight hours after transfection, cells were infected with HSV-2 at 3 MOI or mock-infected. Cell lysates were collected at 0 h, 4 h, 6 h, 16 h post-infection and analyzed by Western blotting with antibodies against p-IRF7, total IRF7 and Actin. Knockdown of TLR4 decreased the phosphorylation of IRF7 by HSV2 infection. The experiment was repeated three times, the representative images from one experiment are shown.

Besides IRF3, IRF7 is another main regulator of TLR-mediated type I IFN response to virus infection [20]. The chromatin structure and promoter architecture of *IFNB* gene have revealed that IFN-β transcriptional activation requires the enhancer region located immediately upstream of the core promoter. Virus infection results in the coordinate activation of NF-κB, ATF-2/c-Jun, IRF3, and IRF7 that assemble sequentially on this IFN-β enhancer region [22], [26]. However, there is discrepancy if IRF7 is required for the induction of IFN-β upon virus infection [20], [21], [22], [23]. We asked whether IRF7 is activated in response to HSV-2 infection in HCE cells and whether TLR4 mediates the virus induced activation of IRF7. HCE cells were transfected with scrambled shRNA control or iTLR4 and then infected with HSV-2 at 3 MOI or mock-infected. The cell lysates were harvested from above cells at the indicated time points post-infection (p.i.) for Western blot analysis. As shown in Figure 3, IRF7 was phosphorylated at 4 h after HSV-2 infection compared to mock-infected control HCE cells. The level of phosphorylated IRF7 reached a peak at 6 h p.i. and then declined at 16 h p.i., while in the HCE cells where TLR4 was knockdown with iTLR4 as described above, IRF7 activation in response to HSV-2 was impaired (Figure 3). We were not able to observe an obvious peak of phospho-IRF7 at 6 h post-infection (Figure 3). During the time-course of viral infection, the total IRF7 remained unchanged (Figure 3) and actin was used as the internal loading control. These data suggested that IRF7 is activated in response to HSV-2 infection and TLR4 mediates the phosphorylation of IRF7 in HCE cells.

### Adaptors, TRIF and TRAM, are involved in TLR4-dependent expression of IFN-β in response to HSV-2

**Figure 4:**
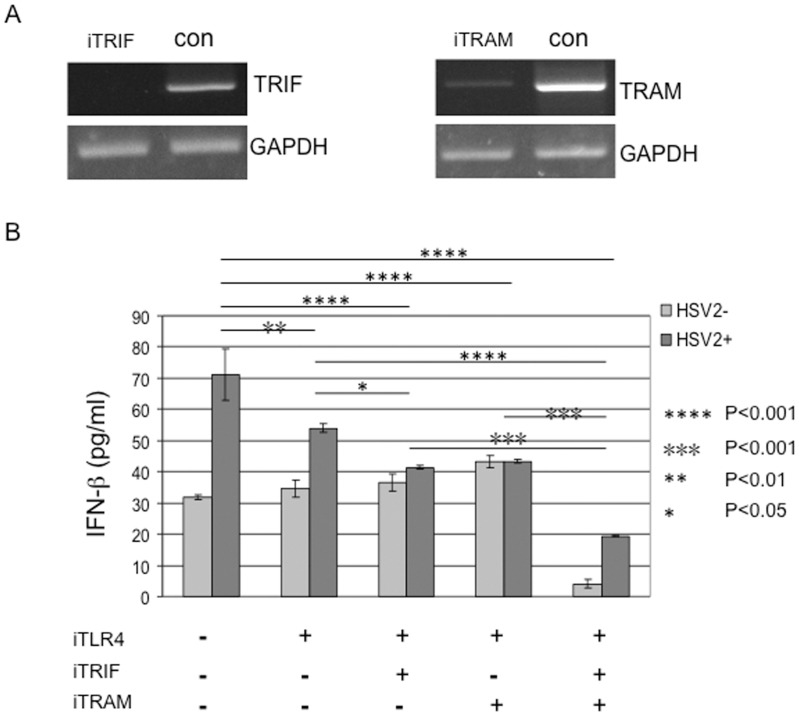
TRIF and TRAM are involved in HSV-2 induced TLR4-dependent production of IFN-β. (A) Confirmation of knockdown of TRIF and TRAM. HCE cells were transfected with scrambled siRNA control or siRNA-TRIF (refer to iTRIF) or siRNA-TRAM (refer to iTRAM). Forty-eight hours after transfection, total RNAs were collected and the expression of TRIF and TRAM was analyzed by RT-PCR. The expression of GAPDH was used as the internal control. The experiment was repeated three times, the representative images from one experiment are shown. (B) HCE cells were transfected with negative control siRNA duplexes or iTLR4 or iTRIF and iTRAM or iTRIF/iTRAM together with iTLR4. At 48 h after transfection, HCE cells were infected with HSV-2 at 3 MOI or mock-infected. Cell-free supernatants were collected at 6 h post-infection for measurement of IFN-β secretion by ELISA analysis. Each assay was performed in triplicate. Results are reported as means ± SD. * indicates p<0.05, ** indicates p<0.01, *** and **** indicates p<0.001.

IRF7 can interact with MyD88 and both of them are required for the induction of type I IFN in mice DCs stimulated by CpG B [20]. Our previous study has found the involvement of adaptors MyD88/Mal in TLR4-dependent signaling in response to HSV-2 in HCE cells [27]. However, another adaptor molecule TRIF has been reported to be able to activates IRF3 and NF-???B, both of which are essential for induction of IFN-β [28]. Therefore we asked if another pair of downstream adaptor molecules TRIF/TRAM is involved in TLR4-dependent signaling in response to HSV-2. Firstly, we confirmed the knockdown efficiency of TRIF and TRAM in HCE cells by siRNA duplexes iTRIF and iTRAM (Figure 4A). Next, we transfected HCE cells with iTRIF, iTRAM or iTRIF/iTRAM, with or without iTLR4. After 48 h of transfection, cells were infected with HSV-2 at 3 MOI or mock-infected. The supernatants were collected at 6 h p.i. and measured for the secretion of IFN-β by ELISA. In Figure 4B, measured IFN-β was 31.80±0.68 pg/ml in supernatant of HCE cells with mock infection and scramble siRNA transfection. HSV-2 infection resulted in a dramatic increase (71.14±8.15 pg/ml) of IFN-β (p<0.001). While knockdown of TLR4 alone decreased the secretion of IFN-β (p<0.01), we observed further reduced expression of IFN-β when knockdown of TLR4 together with either TRIF (p<0.001) or TRAM (p<0.001). More importantly, we showed about 71% decrease in IFN-β production with knockdown of TLR4 together with both TRIF and TRAM (p<0.001). These data indicated that adaptor molecules, TRIF and TRAM, are also involved in TLR4-dependent production of IFN-β in response to HSV-2 in HCE cells.

## Discussion

We have shown for the first time that HSV-2 activates TLR4-dependent NF-kB activation and TLR4-dependent Mal/MyD88/NF-kB signaling contributes to the innate immune response in HCE cells [19], [27]. In this study, we confirmed that HSV-2 infection induces TLR4-dependent expression of IFN-β by knockdown experiment (Figure 1). A recent report also indicated the critical role of TLR4 in mediating innate response during primary infection of human lymphatic endothelial cells (LEC) and lytic replication of the latent Kaposi Sarcoma herpesvirus (KSHV) [29]. TLR9 is thought to be the typically sensor of DNA viruses such as HSV. Studies using natural DNA suggest that both CpG content and the level of methylation of the motif strongly affect the ability of DNA to activate TLR9 [30]. TLR9 is only important for the host immune response to pathogens that can reach the lymphoid organs or blood [18], [31]. The innate immune response to HSV infection is complex since different virus strains have different cellular tropisms and the host immune response to viral infection is also cell-type specific.

**Figure 5:**
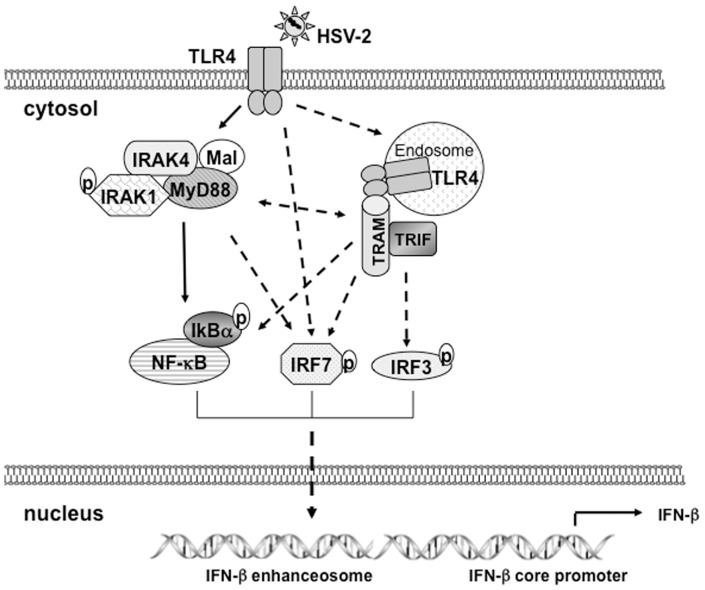
The proposed model that IRF3 and IRF7 are involved in TLR4-dependent induction of IFN-β by HSV-2 in HCE cells. HSV-2 infection induces TLR4 dependent signaling pathways. In addition to MyD88/Mal/IRAK1/NF-kB axis (data in submitted manuscript), adaptors molecules TRIF/TRAM may be activated following virus induced endocytosis. The key regulators IRF3 and IRF7 are phosphorylated and translocated into nucleus, thereby assembling the enhanceosome together with NF-kB and regulating the transcription activation of immediately downstream of IFN-β core promoter. The solid arrow lines represent the experiments confirmed signaling pathways. The dotted arrow lines represent the hypothetic signaling pathways.

Our previous study showed that molecules MyD88 and Mal are required in TLR4-dependent signaling in response to HSV-2 (Figure 5) [27]. Silenced expression of Mal/MyD88 suppressed the production of IL-6, but showed partially reduced effect on the production of IFN-β [27]. Type I IFN is induced only in the intracellular compartments [32]. Induction of the *IFNB* gene by signaling through TLR4 is mostly TRAM-TRIF dependent in macrophages stimulated with LPS [25]. Our data demonstrate that TRIF and TRAM may play a role in TLR4-dependent induction of IFN-β in response to HSV-2 by ELISA analysis (Figure 4). Interestingly, when knockdown both adaptors and TLR4, the basal level of IFN-β was extremely low. We repeated the experiments and observed the similar pattern of IFN-β. We believe that TLR4 and TRIF/TRAM are involved in IFN-β production by HSV-2, while we could not rule out other factors that decrease basal level of IFN-β but are TLR4-unrelated.

Type I IFN is critical for the antiviral activity of the epithelial cells that it is regulated by multiple factors, for example, activation of NF-&B, ATF-2/c-Jun, IRF3, and IRF7 may interact with IFN-β promoter or enhancer region, thereby induce its expression, while other signals lead to separately activation of each transcription factors [22], [26]. Our data demonstrated that TLR4 mediates the phosphorylation and activation of IRF3 (Figure 2), which thereafter regulates the expression of IFN-β in response to HSV-2. The phosphorylation of IRF3 was observed as early as 4 h p.i. and remained the elevated level at 16 h p.i.. In Figure 1, the secretion of IFN-β peaks at 6 h p.i. and declines at 16 h p.i.. The discrepancy between the different time points of phosphorylation of IRF3 and production of IFN-β may due to the other regulators and/or signaling of IFN-β. It is very important that immune response returns to the sensitive threshold level soon after pathogen stimulation.

Different cell types are likely to have their own pathways to induce type I IFN in response to different viruses, for example, IRF8 and IRF3 cooperatively regulate IFN-β induction in human monocytes to respond Sendai Virus [8], [20], [21], [22], [23]. There is discrepancy if IRF7 is required for the induction of IFN-β upon virus infection. Our data demonstrated that IRF7 is phosphorylated and activated in TLR4-dependent innate signaling in response to HSV-2 in HCE cells (Figure 3). We observed the phosphorylation of IRF7 as early as 4 h p.i.. The level of phosphorylated IRF7 reached a peak at 6 h p.i. and then declined at 16 h p.i..This is correlated to the time-course of IFN-β secretion in Figure 1. But still we can not assure whether IRF7 is a finer regulator than IRF3 in regulating expression of IFN-β. Our previous study showed the involvement of adaptors MyD88/Mal in TLR4-dependent signaling in response to HSV-2 [27]. Thus, we propose a work model of how HSV-2 infection induces IFN-β shown in Figure 5. The pathways may include: 1. HSV-2 induces and activates TLR4; 2. MyD88/Mal regulates NF-kB activity; 3. MyD88/Mal and TRAM/TRIF may separately or synergically affects IRF3/IFR7 phosphorylation; 4. TRAM/TRIF may regulate NF-kB activity; 5. TLR4 may directly affect IRF7 phosphorylation; 6. The phosphorylated NF-kB, IRF3 and IRF7 may sequentially or synergically regulate IFN-β expression. It would be important to investigate which adaptor molecule, MyD88 or TRIF or both, mediates the activation of IRF7 and IRF3 in the future study. Currently, we speculate that IRF7, IRF3 and NF-kB are involved, most likely assembling enhanceosome, in regulating the expression of IFN-β in the HCE cells upon HSV-2 infection (Figure 5).

## Materials and Methods

### Cells and virus

Human cervical epithelial (HCE) cells immortalized by hTERT were described in the early studies [19], [33]. HCE cells were grown in keratinocyte serum-free medium (K-SFM) (Invitrogen) supplemented with the provided 50 ug/ml bovine pituitary extract, 0.1 ng/ml recombinant epidermal growth factor, and 1% penicillin/streptomycin (Invitrogen). Vero cells (ATCC) were maintained in complete Dulbecco's modified Eagle's medium (DMEM) supplemented with 10% fetal bovine serum (FBS) and 1% penicillin/streptomycin. Cells were incubated at 37°C in a humidified incubator containing 5% CO_2_. Herpes simplex virus 2 (HSV-2, G strain) was obtained from American Type Culture Collection (ATCC). All stocks of HSV-2 were propagated and titrated using a standard plaque assay in Vero cells. For virus infection, HCE cells were infected with HSV-2 at a multiplicity of infection (MOI) of 3. At the indicated time, cells were lysed for Western blot analysis or luciferase assay, and conditioned media were collected for cytokine measurements by ELISA.

### Antibodies and plasmids

Rabbit polyclonal antibodies against IRF3, IRF7, phospho-IRF3 and phospho-IRF7 were obtained from Cell Signaling Technology (Danvers, MA). Mouse monoclonal antibody against actin was purchased from Santa Cruz Biotechnology (Santa Cruz, CA). The shRNA Construct specific for TLR4 and shRNA pGFP-V-RS Vector were purchased from ORIGENE (Rockville, MD). The siRNA duplexes specific for human TRIF and TRAM and scrambled control siRNA duplexes were obtained from Santa Cruz Biotechnology (Santa Cruz, CA).

### Transfection

Cells were plated in a 6-well plate or 24-well plate one day before transfection and were grown to 50% confluence. Cells were transfected with Lipofectamine 2000 (Invitrogen) and harvested for total RNA or cell-lysate at the indicated time points. The knock-down efficiency of genes was determined by regular RT-PCR.

### Reverse Transcriptase-PCR

Total RNA from 2×10^6^ HCE cells was extracted with RNApre pure cell RNA kit containing DNase I (TIANGEN Biotech, Beijing). Two microgram total RNA was reverse transcribed with random primer (Promega). Specificity of RT-PCR was controlled with no-template as well as no-reverse transcriptase samples. Results are normalized to the housekeeping gene GAPDH. The following primers were used: GAPDH forward: 5′-CTCAGACACCATGGGGAAGGTGA-3′, Reverse: 5′-ATGATCTTGAGGCTGTTGTCATA-3′; TLR4 forward: 5′-GGTCCTCAGTGTGCTTGTAGTA-3′, Reverse: 5′-CAGATAGATGTTGCTTCCTGCC-3′; TRIF forward: 5′- CTGGGTAGTTGGTGCTGGTT -3′, Reverse: 5′- ATTGACGGTGTTTCGGACTG -3′; TRAM forward: 5′- AGGAAAGCAGGAGGGAGC -3′, reverse: 5′- AAGGCATTGATGGTTTGGAG -3′.

### Western blot analysis

At indicated time points, HCE cells were harvested with modified radioimmuno-precipitation assay (RIPA) buffer (50 mM Tris-Cl, 150 mM NaCl, 2 mM EDTA, 1% Nonidet P-40, 0.5% Nadeoxycholate, and 0.1% SDS [pH 7.4]) with protease inhibitors (Sigma). The cell lysates were centrifuged at 15,000 rpm for 20 min at 4°C and the supernatants were collected and a BCA (Beyotime) protein assay was performed. Fifty of total extract was subjected to electrophoresis on 12% SDS-PAGE gel, and transferred onto polyvinylidene difluoride (PVDF) membranes (Millipore). Membranes were blocked in 5% milk-TBST buffer at 4°C. The membranes were probed with specific antibodies described in Antibodies and plasmids. Blots were developed using an enhanced chemiluminescence reagent (Beyotime).

### Enzyme-linked immunosorbent assay (ELISA)

HCE cells were cultured at 1×10^4^ cells/well in flat-bottom 24-well plates for 16–24 h, transient transfected with scrambled control siRNA duplexes or siRNA duplexes targeting for TRIF and TRAM (refer to iTRIF or iTRAM), with or without shRNA-TLR4. At 48 h after transfection, HCE cells were infected with HSV-2 at 3 MOI. Cell-free supernatants were collected at 6 hrs p.i. for ELISA analysis. The secretion of IFN-β was measured according to the manufacturer's instructions (R&D Systems, Abingdon, UK). The optical density was measured using a Bio-Kinetics microplate reader (Bio-Tek Instruments). Each assay was performed in triplicate. Results are reported as means ± SD.

### Statistical analysis

Two-way ANOVA, followed by the Bonferroni posttest, was used for analysis of the data by software Prism 5.0 (GraphPad Software, La Jolla, CA). For each test, differences were considered significant at p<0.05, and data are shown as mean ± SD. All of the experiments were reproducible and carried out in duplicate. Each set of experiments was repeated at least three times.

## References

[1] Freeman EE, Weiss HA, Glynn JR, Cross PL, Whitworth JA, et al (2006) Herpes simplex virus 2 infection increases HIV acquisition in men and women: systematic review and meta-analysis of longitudinal studies. Aids 20: 73–83. 16327322 10.1097/01.aids.0000198081.09337.a7

[2] Tang Q, Qin D, Lv Z, Zhu X, Ma X, et al (2012) Herpes simplex virus type 2 triggers reactivation of Kaposi's sarcoma-associated herpesvirus from latency and collaborates with HIV-1 Tat. PLoS One 7: e31652. 22347501 10.1371/journal.pone.0031652 PMC3276581

[3] Corey L, Bodsworth N, Mindel A, Patel R, Schacker T, et al (2007) An update on short-course episodic and prevention therapies for herpes genitalis. Herpes 14 Suppl 1 5A–11A. 17877886

[4] Chan T, Barra NG, Lee AJ, Ashkar AA (2011) Innate and adaptive immunity against herpes simplex virus type 2 in the genital mucosa. J Reprod Immunol 88: 210–218. 21334750 10.1016/j.jri.2011.01.001

[5] Zhao X, Deak E, Soderberg K, Linehan M, Spezzano D, et al (2003) Vaginal submucosal dendritic cells, but not Langerhans cells, induce protective Th1 responses to herpes simplex virus-2. J Exp Med 197: 153–162. 12538655 10.1084/jem.20021109 PMC2193810

[6] MacDonald EM, Savoy A, Gillgrass A, Fernandez S, Smieja M, et al (2007) Susceptibility of human female primary genital epithelial cells to herpes simplex virus, type-2 and the effect of TLR3 ligand and sex hormones on infection. Biol Reprod 77: 1049–1059. 17881767 10.1095/biolreprod.107.063933

[7] Nazli A, Yao XD, Smieja M, Rosenthal KL, Ashkar AA, et al (2009) Differential induction of innate anti-viral responses by TLR ligands against Herpes simplex virus, type 2, infection in primary genital epithelium of women. Antiviral Res 81: 103–112. 19013198 10.1016/j.antiviral.2008.10.005

[8] Yao XD, Rosenthal KL (2011) Herpes simplex virus type 2 virion host shutoff protein suppresses innate dsRNA antiviral pathways in human vaginal epithelial cells. J Gen Virol 92: 1981–1993. 21632561 10.1099/vir.0.030296-0

[9] Sato A, Linehan MM, Iwasaki A (2006) Dual recognition of herpes simplex viruses by TLR2 and TLR9 in dendritic cells. Proc Natl Acad Sci U S A 103: 17343–17348. 17085599 10.1073/pnas.0605102103 PMC1859932

[10] Lund J, Sato A, Akira S, Medzhitov R, Iwasaki A (2003) Toll-like receptor 9-mediated recognition of Herpes simplex virus-2 by plasmacytoid dendritic cells. J Exp Med 198: 513–520. 12900525 10.1084/jem.20030162 PMC2194085

[11] Kurt-Jones EA, Belko J, Yu C, Newburger PE, Wang J, et al (2005) The role of toll-like receptors in herpes simplex infection in neonates. J Infect Dis 191: 746–748. 15688290 10.1086/427339

[12] Lund JM, Linehan MM, Iijima N, Iwasaki A (2006) Cutting Edge: Plasmacytoid dendritic cells provide innate immune protection against mucosal viral infection in situ. J Immunol 177: 7510–7514. 17114418 10.4049/jimmunol.177.11.7510

[13] Finberg RW, Knipe DM, Kurt-Jones EA (2005) Herpes simplex virus and toll-like receptors. Viral Immunol 18: 457–465. 16212524 10.1089/vim.2005.18.457

[14] Hochrein H, Schlatter B, O'Keeffe M, Wagner C, Schmitz F, et al (2004) Herpes simplex virus type-1 induces IFN-alpha production via Toll-like receptor 9-dependent and -independent pathways. Proc Natl Acad Sci U S A 101: 11416–11421. 15272082 10.1073/pnas.0403555101 PMC509215

[15] Krug A, Luker GD, Barchet W, Leib DA, Akira S, et al (2004) Herpes simplex virus type 1 activates murine natural interferon-producing cells through toll-like receptor 9. Blood 103: 1433–1437. 14563635 10.1182/blood-2003-08-2674

[16] Sato A, Iwasaki A (2004) Induction of antiviral immunity requires Toll-like receptor signaling in both stromal and dendritic cell compartments. Proc Natl Acad Sci U S A 101: 16274–16279. 15534227 10.1073/pnas.0406268101 PMC528964

[17] Ferreira VH, Nazli A, Mossman KL, Kaushic C (2013) Proinflammatory cytokines and chemokines - but not interferon-beta - produced in response to HSV-2 in primary human genital epithelial cells are associated with viral replication and the presence of the virion host shutoff protein. Am J Reprod Immunol 70: 199–212. 23621693 10.1111/aji.12133

[18] Paludan SR, Bowie AG, Horan KA, Fitzgerald KA (2011) Recognition of herpesviruses by the innate immune system. Nat Rev Immunol 11: 143–154. 21267015 10.1038/nri2937 PMC3686362

[19] Li H, Li X, Wei Y, Tan Y, Liu X, et al (2009) HSV-2 induces TLRs and NF-kappaB-dependent cytokines in cervical epithelial cells. Biochem Biophys Res Commun 379: 686–690. 19124006 10.1016/j.bbrc.2008.12.150

[20] Honda K, Yanai H, Negishi H, Asagiri M, Sato M, et al (2005) IRF-7 is the master regulator of type-I interferon-dependent immune responses. Nature 434: 772–777. 15800576 10.1038/nature03464

[21] Dai P, Cao H, Merghoub T, Avogadri F, Wang W, et al (2011) Myxoma virus induces type I interferon production in murine plasmacytoid dendritic cells via a TLR9/MyD88-, IRF5/IRF7-, and IFNAR-dependent pathway. J Virol 85: 10814–10825. 21835795 10.1128/JVI.00104-11 PMC3187486

[22] Sin WX, Li P, Yeong JP, Chin KC (2012) Activation and regulation of interferon-beta in immune responses. Immunol Res 53: 25–40. 22411096 10.1007/s12026-012-8293-7

[23] Li P, Wong JJ, Sum C, Sin WX, Ng KQ, et al (2011) IRF8 and IRF3 cooperatively regulate rapid interferon-beta induction in human blood monocytes. Blood 117: 2847–2854. 21228327 10.1182/blood-2010-07-294272

[24] Peng T, Zhu J, Klock A, Phasouk K, Huang ML, et al (2009) Evasion of the mucosal innate immune system by herpes simplex virus type 2. J Virol 83: 12559–12568. 19793807 10.1128/JVI.00939-09 PMC2786730

[25] Honda K, Taniguchi T (2006) IRFs: master regulators of signalling by Toll-like receptors and cytosolic pattern-recognition receptors. Nat Rev Immunol 6: 644–658. 16932750 10.1038/nri1900

[26] Apostolou E, Thanos D (2008) Virus Infection Induces NF-kappaB-dependent interchromosomal associations mediating monoallelic IFN-beta gene expression. Cell 134: 85–96. 18614013 10.1016/j.cell.2008.05.052

[27] Liu H, Chen K, Feng W, Wu X, Li H (2013) TLR4-MyD88/Mal-NF-kB axis is involved in infection of HSV-2 in human cervical epithelial cells. PLoS One 8: e80327. 24278275 10.1371/journal.pone.0080327 PMC3835891

[28] Fitzgerald KA, McWhirter SM, Faia KL, Rowe DC, Latz E, et al (2003) IKKepsilon and TBK1 are essential components of the IRF3 signaling pathway. Nat Immunol 4: 491–496. 12692549 10.1038/ni921

[29] Lagos D, Vart RJ, Gratrix F, Westrop SJ, Emuss V, et al (2008) Toll-like receptor 4 mediates innate immunity to Kaposi sarcoma herpesvirus. Cell Host Microbe 4: 470–483. 18996347 10.1016/j.chom.2008.09.012 PMC2698447

[30] Yasuda K, Richez C, Uccellini MB, Richards RJ, Bonegio RG, et al (2009) Requirement for DNA CpG content in TLR9-dependent dendritic cell activation induced by DNA-containing immune complexes. J Immunol 183: 3109–3117. 19648272 10.4049/jimmunol.0900399 PMC2860771

[31] Takeuchi O, Akira S (2010) Pattern recognition receptors and inflammation. Cell 140: 805–820. 20303872 10.1016/j.cell.2010.01.022

[32] Kagan JC, Su T, Horng T, Chow A, Akira S, et al (2008) TRAM couples endocytosis of Toll-like receptor 4 to the induction of interferon-beta. Nat Immunol 9: 361–368. 18297073 10.1038/ni1569 PMC4112825

[33] Miller J, Dakic A, Chen R, Palechor-Ceron N, Dai Y, et al (2013) HPV16 E7 protein and hTERT proteins defective for telomere maintenance cooperate to immortalize human keratinocytes. PLoS Pathog 9: e1003284. 23592995 10.1371/journal.ppat.1003284 PMC3617164

